# Gun-Shot Wound of the Chest*Read before Central New York Medical Association, at Syracuse, N. Y., November 15, 1887.

**Published:** 1888-01

**Authors:** J. Van Duyn

**Affiliations:** Syracuse, N. Y.


### *GUN-SHOT WOUND OF THE CHEST, INVOLVING THE BRACHIAL-PLEXUS, AXILLARY ARTERY, ATTENDED WITH NEURITIS AND ANEURISM, LIGATURE OF SUBCLAVIAN ARTERY AND RECOVERY.**

By Dr. J. VAN DUYN, Syracuse, N. Y.

The following case, which I have to report, is one of gun-shot wound of the chest, involving the lung, the brachial-plexus and the axillary artery, followed by neuritis and aneurism, ligature of the subclavian artery and recovery.

O. B., October 17th, was shot, in the afternoon of May 22, 1886, while seated in a buggy trying to control a fractious horse. The ball, which was round, three-eighths inch in diameter, was one of nine fired from a shot-gun, at a distance of about six rods. It passed th rough the wagon-top, and entered theback one and one-half inch to the right of the eighth dorsal spine, and, as was afterwards determined by the location of the ball after it had lodged, it took a direction forwards, upward and little outwards, to a point directly under the middle of the right clavicle. This direction was possible, because of the position of the boy leaning forward when shot.

After the injury, there was immediate loss of power in the right hand, and the rein was dropped. I saw him half an hour afterwards, and found him with all the evidences of severest shock. He was pale, bathed in a cold sweat, almost pulseless, extremely exhausted, and incoherent in his utterances. Only the wound of entrance of the ball in the back could be found, which was bulging and crepitant, showing that the lung had been injured, and from it air had escaped along the track of the wound, and filled the tissues in its vicinity. Late in the evening, a swelling was found under the middle of the right clavicle, which was pointed, partly firm and partly compressible and crepitant. Until this time, there had been but little cough, without expectoration. Deep respiration now caused pain, not

*Read before Central New York Medical Association, at Syracuse, N. Y., November 15, 1887.

only in the chest, but also in the right shoulder and elbow. The first and second fingers had become numb at their extremities. Immediately on seeing him, I administered brandy and morphine, which were repeated twice in the evening, and at eight o'clock, P. M., he slept. At eleven o'clock, P. M., he vomited, with great force, large quantities of solid and liquid matter, after which he slept. On the following day, at five o'clock, A. M., he coughed up a small blood-clot, and a number of small clots of blood in the afternoon. Through the day, there was pain in the chest, mental wandering, pain also in arm and back, and great restlessness. On the third day, there was great dyspnœa and excessive heart's action. Although auscultation had been practiced from the first to determine the condition of the lung, which manifested itself by an almost entire absence of respiratory movement and no rales, no sound connected with the circulation had been discovered. But now, over the tumor, under the clavicle, there was a rough sound, synchronous with the cardiac systole. From this time on, the dyspnœa gradually improved, and the normal vesicular respiration sound reappeared in the wounded lung. The percussion note became more resonant as low as the fourth and fifth rib, where, until then, it had been flat. The tumor under the clavicle was less prominent, and there was increasing distinctness in the determination of a foreign body, which could be no other than the ball. In front, crepitation was lost in the swelling, but behind, at wound of entrance, it persisted. The pain in the arm was complained of continually, on the radial side; from the elbow downwards into the thumb and first and second fingers. Both fingers were numb, but, over the area of pain in the arm and thumb, there was marked hyperæsthesia. The first temperature record was made on the morning after the injury, when it was 101.5°, rising in the evening to 102°. The temperature fell on the fourth day, and from that time until the end of the record, never rose again above 100.5°. On June 6th, fifteen days after the injury, no crepitation could be felt in the wound in the back. Condition greatly improved, but patient complained of the pain in the right arm. On June 14th, twenty-three days after injury,

I removed the ball from where it had long been felt, under the middle of the right clavicle. To-day, Dr. Breed made tracings of the pulses of both radials. To the finger, that of the right side was the feeble, and seemed to have a different character from the left. The tracing by the sphygmograph, however, shows no essential difference, and represents only a frequent, dicrotic pulse on both sides. This pulse trace corresponds almost exactly with that of October 11th. After removal of the ball, there was no swelling left under the clavicle. June 19th, I made the following observation on the effect of the nerve injury. The pain in the arm, from the elbow down, very severe, and alternates with sensation of burning. The temperature in the web between the first finger and thumb of the right hand was 97.8°; of the left hand, 98.4°. The thermometer grasped in the carpo-metocarpal joints of the ulna side of the right hand showed 98.3°, or .5° higher on this than on the other side of the same hand. The temperature under the tongue was 98.7°. Pulse, 104°; respiration, 18°. The finger-nails had not been cut since the injury, because of the pain which the attempt to cut those of the thumb and first finger produced, and now, twenty-eight days afterwards, the free ends of the nails of the ring and little fingers were three times longer than those of the thumb and index fingers. Besides, the palmar skin of the thumb and first finger was horny and separating in flakes, while that of the ring and little fingers was soft, smooth and unbroken. It was interesting to note that the middle finger shared in the condition of both sides of the hand. For the two days following this examination, the pain in the right fore-arm and hand, alternating with burning, was so severe as to prevent all but one hour's sleep in forty-eight hours. The temperature under the tongue rose to 100.5°. June 24th—Has no sensation of burning during the past thirty-six hours, but little pain in the day-time, pain more or less severe at night. On July 1st, the young man went to the Thousand Islands, and remained there until October 1st. During this stay on the St. Lawrence, pain and burning in the arm and hand was felt only during damp, rainy weather. But there was added a new sensation: a numbness of the whole right hand, or of a part of it, at one time of one region, and at another time of another region; which, with some intermission, became quite continuous. This may be explained by the fact of a tumor, which was first noticed in July, showing itself where the ball had been cut out under the middle of the right clavicle. When he returned October 1st, I found this tumor to be quite prominent, smooth, distinctly fluctuating, and having the impulse of the artery behind it. Over the tumor, the pulse was heard with a rasping sound. Pressure on the tumor produced severe pain and pricking in fore-arm and hand, in the exact region where the pain and burning had been felt before. This tumor, now known to be an aneurism, connected with the axillary artery, explained the numbness which began to be felt in July, as well as the rasping bruit which was heard here since the second day after the injury. On October 10th, when we made an examination, we found the arm practically useless. The reaction to faradism was lost in the radial group of muscles, except feebly to the strongest currents, and all the remaining muscles of the fore-arm and hand reacted but feebly to ordinary currents. Anæsthesia was clearly limited to the radial side of the fore-arm and hand. Here we had, doubtless, the double effects of the original injury to the brachial-plexus, and of the presence due to the growth of the tumor. I exhibit the pulse traces kindly taken by Dr. Breed. These show none of the signs of aneurism, but rather of a great loss of arterial tension, frequent heat action, and quickness of pulse current. The right arm, fore-arm and hand were atrophied. The circumference of the right arm was 21½ cm.; of the left, 24¼ cm.; of the right fore-arm, 22 cm.; of the left, 24 cm.

*November 2d*—Although the patient was able to sit up, move about and go to his meals in the fourth week after he was wounded, and had been able to spend three months at the Thousand Islands in semi-activity, yet, from the first shock, he had never regained his vigor. He was tall for his age, flabby, weak, and had an over-active heart and feeble pulse. There was no question as to the necessity of surgical interference for the cure of aneurism, which, if left uncured, would destroy his life. But, under these conditions of general vigor and condition of tissues, an operation of the magnitude of that of ligature of the subclavian artery was to be thought of only with forebodings of a bad result. On this date, I determined to confine him to bed, hoping that, by rest and feeding, his condition would improve. Accordingly, he went to bed in a quiet part of the house, avoided all company, and led a life of quiet vegetation. On the second day thereafter, November 4th, examination showed he had quite lost the power to abduct the arm, rotate the fore-arm, and extend the fingers. He was kept in bed, where he greatly improved in all respects continuously until January 17th of the present year, eight months after the injury, when it seemed best to operate. Accordingly, in the presence of Drs. Dunlap, Mercer, Breese, Loomis and Hinsdale, the patient having been anæsthetized by ether, I ligated the subclavian artery with double gut ligature, cut short, on the first rib. The operation was performed in the usual manner, as directed in the books, with the ordinary antiseptic precautions, without the spray. The wound was closed by eight chromicized cat-gut sutures, without drainage, and was covered by gauze filled with iodoform. On the third day, I removed the dressings, and found that there had been but little extravasation from the wound, and that wound and stitches were dry. Union was found firm and complete, and I removed the stitches. During the performance of the operation, all went smoothly, except that while forcing my way on the scalenus down to the artery, and when within a considerable distance above the artery, the tissues suddenly gave way, and my finger entered a large open cavity. This was investigated, as far as it was thought safe, but without any conclusion being reached as to the origin of this space, which was finally disregarded, and the operation proceeded with. In the morning, after operation, the temperature rose to 101.4°, but fell in the afternoon to 99.4°. There was no subsequent rise above 100°. On January 26th, nine days after the operation, the thermometer, grasped in the right hand, showed 98°; by the left hand, 98.5°, while the temperature under the tongue was 99.5°. On January 29th, I could count the pulse of the right wrist, but with the greatest difficulty, because it could be perceived only with the nicest condition of pressure. After the operation, all numbness in fore-arm and hand disappeared, but could be reproduced by pressure on the aneurism. This could be produced also by pressure anterior to elbow in middle line, the numbness and pricking even affecting the end of second finger. On February 13th, a testing of the length of the finger-nails showed no difference in their growth since the operation. The temperature of the hands was found equal. March 21st—Is rapidly regaining his strength and vigor. At times, the right hand becomes numb while he is walking, but the numbness disappears at once on supporting the hand. Having just come in from a temperature of 37°, the right hand is felt to be quite cold objectively, but the left is warm. He says he does not feel the difference, and knows of it only by bringing the hand in contact with some other part of the body. The aneurismal tumor smaller. Until he had been in the room sufficiently long to get warm, I could not perceive the pulse in the right radial to count it, but as the hand became warmer, the pulse became distinct, and could be counted. There remains a woolly feeling on radial side of index-finger and inner side of last phalanx of the thumb. The strength of the grasp of the right hand is marked by 80° of the dynamometer scale, but the instrument is not stiff enough to record the strength of the left hand. September 5th—The strength of the right hand cannot be measured by my dynamometer, but there is a marked clumsiness in the functions of the right hand, and there remains the slightest fuzziness in thumb and first finger. The circumference of the left fore-arm is greater than that of the right by 1.9 cm., and the temperature of the left hand is .7° higher. The only other condition which I shall speak of, and which is worthy of note, is that of the eyes. While being taken home after the injury, he was seized by an attack of blindness, consciousness remaining, which lasted but a few moments. A second such attack occurred at the dinner-table, five weeks after the first. After this, they followed in quick succession, or often sometimes as twice in a day, but, generally, twice in a week, until the operation, after which they did not occur. After the first blind attack, he was troubled by floating bodies in the eyes, which, at the time of last consultation, one month ago, had not disappeared. Vision was $\frac{20}{20}$, and no abnormal condition was revealed by ophthalmoscopic examination. The cause of this is not clear. It is evident that the blindness for the time being, and the floating bodies, had a common origin in the wound of the brachial flexus, and that the immediate effect of the injury to the nerve on the vascular supply of the eye, was continued by the pressure of the growing aneurism on the nerve, for, as soon as this pressure was relieved by the ligation of the subclavian artery, the recurring vaso-motor spasm of the eye ceased.

Besides, the many points in the history connected with lung, artery and nerve injury, which, for want of time, I must refrain from reviewing, I call attention to the operation itself, to the contrast between the operation of to-day and that of twenty-five years ago, at the time of the war. Then it fell to my lot to witness an operation for a similar injury. It was performed according to the rules then enforced, the wound was dressed with water, the long silk ligature hung out of the wound, which suppurated, and the soldier died of pleurisy and exhaustion. In the present case, there was no suppuration, no suppurating constriction of the vessel, and, as far as the operation was concerned, all was well in three days. The statistics of operation have entered on a new series, and this record is offered for its proper place and value.

